# Depression and hepatobiliary diseases: a bidirectional Mendelian randomization study

**DOI:** 10.3389/fpsyt.2024.1366509

**Published:** 2024-03-26

**Authors:** Yu Kong, Zhongcai Yao, Lingli Ren, Liqin Zhou, Jinkai Zhao, Yuanyuan Qian, Dayong Lou

**Affiliations:** ^1^ Wenzhou Medical University, Wenzhou, Zhejiang, China; ^2^ Zhuji Hospital Affiliated of Wenzhou Medical University, Shaoxing, Zhejiang, China; ^3^ Basic Medical College, Zhejiang Chinese Medical University, Hangzhou, Zhejiang, China

**Keywords:** depression, hepatobiliary diseases, Mendelian randomization, multivariate Mendelian randomization, univariate Mendelian randomization

## Abstract

**Background:**

More and more evidence suggests a close association between depression and hepatobiliary diseases, but its causal relationship is not yet clear.

**Method:**

Using genome-wide association studies (GWAS) to summarize data, independent genetic variations associated with depression were selected as instrumental variables. Firstly, we designed a univariate Mendelian randomization (UVMR) analysis with two samples and simultaneously conducted reverse validation to evaluate the potential bidirectional causal relationship between depression and various hepatobiliary diseases. Secondly, we conducted a multivariate Mendelian randomization (MVMR) analysis on diseases closely related to depression, exploring the mediating effects of waist to hip ratio, hypertension, and daytime nap. The mediating effects were obtained through MVMR. For UVMR and MVMR, inverse variance weighted method (IVW) is considered the most important analytical method. Sensitivity analysis was conducted using Cochran’Q, MR Egger, and Leave-one-out methods.

**Results:**

UVMR analysis showed that depression may increase the risk of non-alcoholic fatty liver disease (OR, 1.22; 95% CI, 1.03-1.46; *p*=0.0248) in liver diseases, while depression does not increase the risk of other liver diseases; In biliary and pancreatic related diseases, depression may increase the risk of cholelithiasis (OR, 1.26; 95% CI, 1.05-1.50; *p*=0.0120), chronic pancreatitis (OR, 1.61; 95% CI, 1.10-2.35; *p*=0.0140), and cholecystitis (OR, 1.23; 95% CI, 1.03-1.48; *p*=0.0250). In addition, through reverse validation, we found that non-alcoholic fatty liver disease, cholelithiasis, chronic pancreatitis, cholecystitis, or the inability to increase the risk of depression (*p*>0.05). The waist to hip ratio, hypertension, and daytime nap play a certain role in the process of depression leading to non-alcoholic fatty liver disease, with a mediating effect of 35.8%.

**Conclusion:**

Depression is a susceptibility factor for non-alcoholic fatty liver disease, and the causal effect of genetic susceptibility to depression on non-alcoholic fatty liver disease is mediated by waist-hip ratio, hypertension, and daytime nap.

## Introduction

Depression is the leading cause of disability worldwide and is often fatal. Patients with depression exhibit changes in various key functions, including emotions, sleep, and appetite (1, 2). Depression mainly affects people with chronic diseases and cognitive impairments, especially the elderly. Depression increases the pain of these patients, leading to family breakdown and disability, worsening underlying diseases, increasing mortality rates, and imposing a heavy burden on healthcare and socio-economic development (3, 4). An increasing number of studies indicate a close correlation between depression and liver disease, with important roles played by gut microbiota, inflammation, neurotransmitters, and lifestyle habits (5–7). A logistic regression analysis of 1021 patients with liver cirrhosis found that nearly one sixth of them suffered from moderate to severe depression, and nearly half of them suffered from moderate to severe anxiety (8). In addition, a meta-analysis of 7 observational studies (six cross-sectional studies and one cohort study), including a total population of 61617, confirmed a high correlation between non-alcoholic fatty liver disease and depression. However, since most of the articles included in the study were cross-sectional studies, clear causal relationships could not be elucidated (9). For gallbladder disease, studies have shown a possible association between depression and gallbladder. A retrospective cohort study of 6755 gallstone patients found an association between female cholecystectomy and postoperative depression risk, but not among males (10). In addition, hepatocellular carcinoma and cholangiocarcinoma are often associated with significant psychological burdens such as anxiety, depression, and a decline in health-related quality of life, which may lead to psychiatric comorbidities (11). Although numerous studies have shown a causal relationship between depression and non-alcoholic fatty liver disease (12–14), the impact of depression on various hepatobiliary diseases has not been clearly elucidated.

Mendelian randomization is a causal inference method that falls between traditional pathology and randomized controlled studies. Its principle mainly relies on the influence of randomly assigned genotypes in nature on phenotype to infer the impact of biological factors on diseases (15–17). In Mendelian randomization analysis, it is necessary to obtain data from GWAS, identify gene variation SNPs related to biological factors, namely “instrumental variables”, and confirm the causal relationship between exposure factors and outcome variables by excluding confounding factors and reverse relationships, thereby improving the reliability of causal inference (18, 19). Therefore, we conducted an MR study to elucidate the potential bidirectional causal relationship between depression and 18 hepatobiliary diseases. To reveal possible mechanistic pathways, we further conducted multivariate MR analysis to examine the mediating effects of waist to hip ratio, hypertension, and daytime nap.

## Materials and method

### Study design

**Figure 1:**
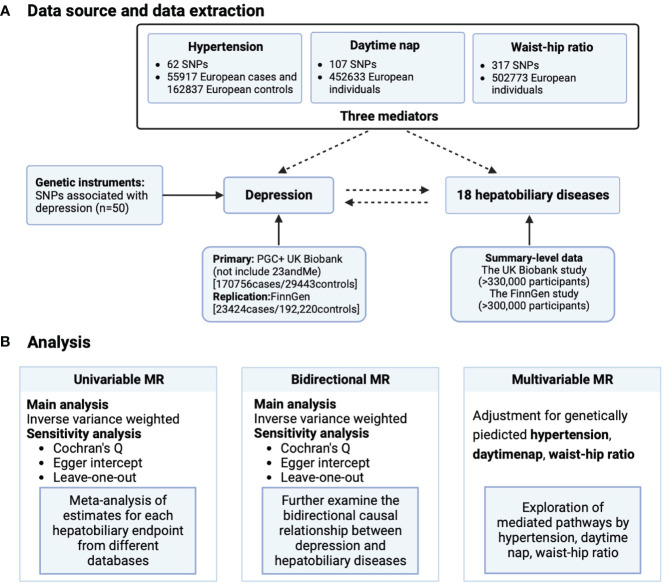
Schematic overview of study design. **(A)** Data source and data extraction and **(B)** analysis. MR, Mendelian randomization; SNP, single nucleotide polymorphisms.

This study is based on publicly available GWAS (**Supplementary Table S1**). MR must satisfy three core hypotheses: (1) correlation hypothesis: instrumental variables are closely related to exposure phenotype; (2) Exclusivity hypothesis: instrumental variables are not related to diseases; (3) Independence assumption: instrumental variables are not related to confounding factors. This MR analysis is conducted from three directions (**Figure 1**): (1) evaluating the impact of depression on various hepatobiliary diseases through univariate MR; (2) Evaluate the impact of various hepatobiliary diseases on depression through reverse MR imaging; (3) Evaluate the mediating effects of waist hip ratio, hypertension, and nap on depression and various hepatobiliary diseases through multivariate MR.

### Instrumental variable selection

We extracted genetic instrumental variables related to depression from the latest GWAS (**Supplementary Table S1**), which included a meta-analysis of 200199 individuals (170756 depression cases and 29443 controls) from the UK Biobank and PGC (excluding 23andme) databases. Additionally, the validation dataset was from FinnGen, which included 23424 cases and 192220 controls. Selection criteria for instrumental variables: (1) the genome-wide significance threshold *p*<5×10^−8^; (2) SNPs with linkage disequilibrium r^2^>0.01 at a window size of 10,000 kb were excluded; (3) SNPs associations with exposure factors were evaluated using the F statistic (F=β^2^/se^2^), and low-statistical-power SNPs will be excluded F<10. No proxy SNPs were used in MR analysis. Finally, 50 SNPs associated with depression were identified as instrumental variables.

### Hepatobiliary diseases data sources

Regarding non-alcoholic fatty liver disease, the most comprehensive summary level data of GWAS was obtained from the meta-analysis of UK Biobank, Estonian Biobank, eMERGE, and FinnGen, with a total of 8434 NAFLD cases and 770180 controls (20). Regarding cholecystitis and chronic pancreatitis, meta-analysis was conducted by UK Biobank and FinnGen, combined with 220 deep phenotypes of GWAS from Japan Biobank (21). Malignant tumors of the liver and intrahepatic bile ducts, cirrhosis, chronic hepatitis, acute pancreatitis, alcoholic liver disease, alcohol induced chronic pancreatitis, and viral hepatitis all come from the FinnGen database. Meta analysis of autoimmune hepatitis, hepatobiliary carcinoma, and chronic hepatitis C originating from UK Biobank and FinnGen; A meta-analysis was conducted on the dataset discovered by GWAS for primary biliary cholangitis, consisting of 2764 subjects with European ancestry and 10475 controls (22). The data on bile duct stones is sourced from UK Biobank, diagnosed according to the ICD10 standard (K80.5 bile duct stones without cholangitis or cholecystitis), including 1706 cases and 461304 controls; Secondary liver malignant tumors originated from UK Biobank and were diagnosed according to ICD10 criteria (C78.7 secondary liver malignant tumors), including 1139 cases and 461871 controls. Alcohol related hepatocellular carcinoma (AHCC) originates from GWAS and consists of 2107 patients aged 20-92 with alcohol related liver disease.

### Data sources for possible mediators

Depression patients have an increased risk of developing metabolic syndrome (MetS), which is a cluster of cardiovascular risk factors including dyslipidemia, abdominal obesity, hypertension, and hyperglycemia (23). Patients with depression typically experience varying degrees of circadian rhythm changes and disturbances in steady-state sleep regulation, all of which are involved in regulating daytime energy and drowsiness levels (24). In addition, previous studies have shown a close association between waist-hip ratio (25), hypertension (26), and daytime nap (27) with hepatobiliary diseases. Therefore, we believe that these three factors play a potential mediating role. Hypertension comes from the FinnGen database, which includes 55917 cases and 162837 controls; The nap utilized the complete British Biobank dataset of European ancestors, including relevant individuals (n=52633) and independent replicated samples from participants in the 23 and Me study of European ancestors (n=541333); For waist to hip ratio, from UKBiobank.

### Statistical analysis

In this study, MR analysis was mainly performed using the IVW method, which assumes the absence of mean pleiotropy effects, making it the most effective method (28). For the independence hypothesis, we believe that waist to hip ratio, hypertension, and napping are the main confounding factors for the association between depression and various liver diseases. The exclusivity assumption is to further eliminate SNPs related to outcomes (*p*>5×10^−5^).

For preliminary analysis, use the Wald ratio method to calculate the exposure effect values for each SNP, and combine them using the IVW method to obtain MR estimates. To ensure the accuracy of the results, we also used two additional methods: Weighted media (29) and MR-Egger (30). Subsequently, we conducted sensitivity analysis using MR Egger intercept test and Cochran Q statistic to test for evidence of level pleiotropy and heterogeneity, respectively. We removed each relevant SNP one by one and calculated the merging effect of the remaining SNPs through Leave on out analysis to evaluate the impact of each SNP on metabolites (30, 31).

**Figure 2:**
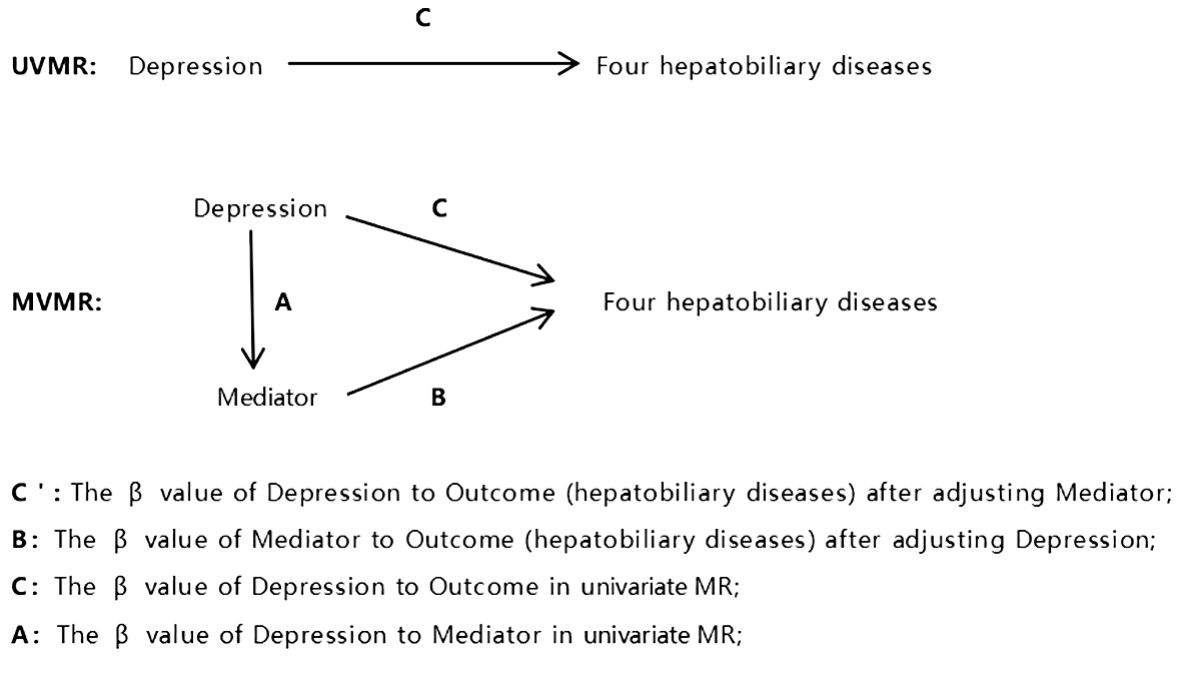
Schematic diagram of modeling methods for mediating effects.

In order to investigate the possible pathways linking depression and hepatobiliary diseases, we used multivariate MR analysis to investigate the significant findings in univariate MR. We first used the IVW method to obtain estimates of the MR effects of depression on each mediator, labeled as A. Then, we used the MVMR method to determine the corrected direct effects (labeled as C ‘) and mediating effects (labeled as B). Multiply the estimated values of A and B for each liver and gallbladder disease as a mediating effect. Finally, for a single mediator, “mediation ratio=(A*B)/C” is used to calculate the proportion of each mediator factor in the total effect. We also use “(C- C ‘)/C” to calculate the ratio of the mediating effect of three media combinations to the total effect (**Figure 2**).

All the above processes were performed in R 4.3.1 using R packages “TwoSampleMR” (32) and “Mendelian randomization” (33) for MR and sensitivity analysis.

## Results

### Analysis of the bidirectional causal relationship between depression and hepatobiliary diseases

**Figure 3:**
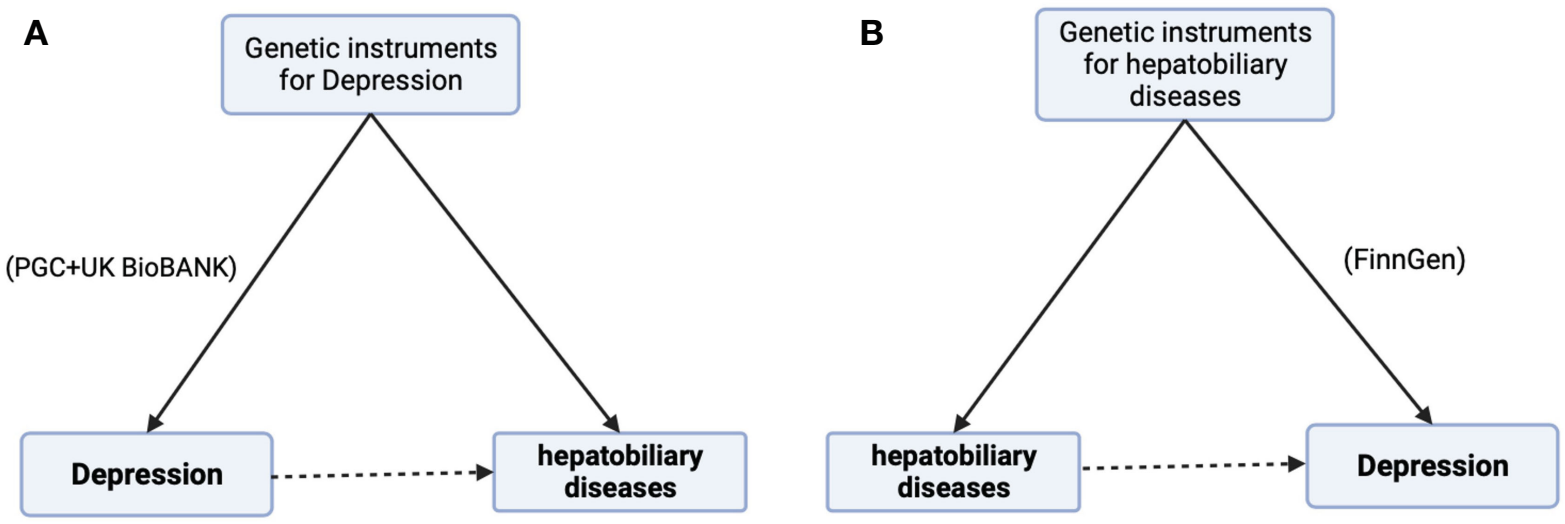
Bidirectional MR estimate. **(A)** Major Depression on 18 hepatobiliary diseases risk and **(B)** Four hepatobiliary diseases where major depression increases risk of morbidity, further explore the reverse effects.

**Figure 4:**
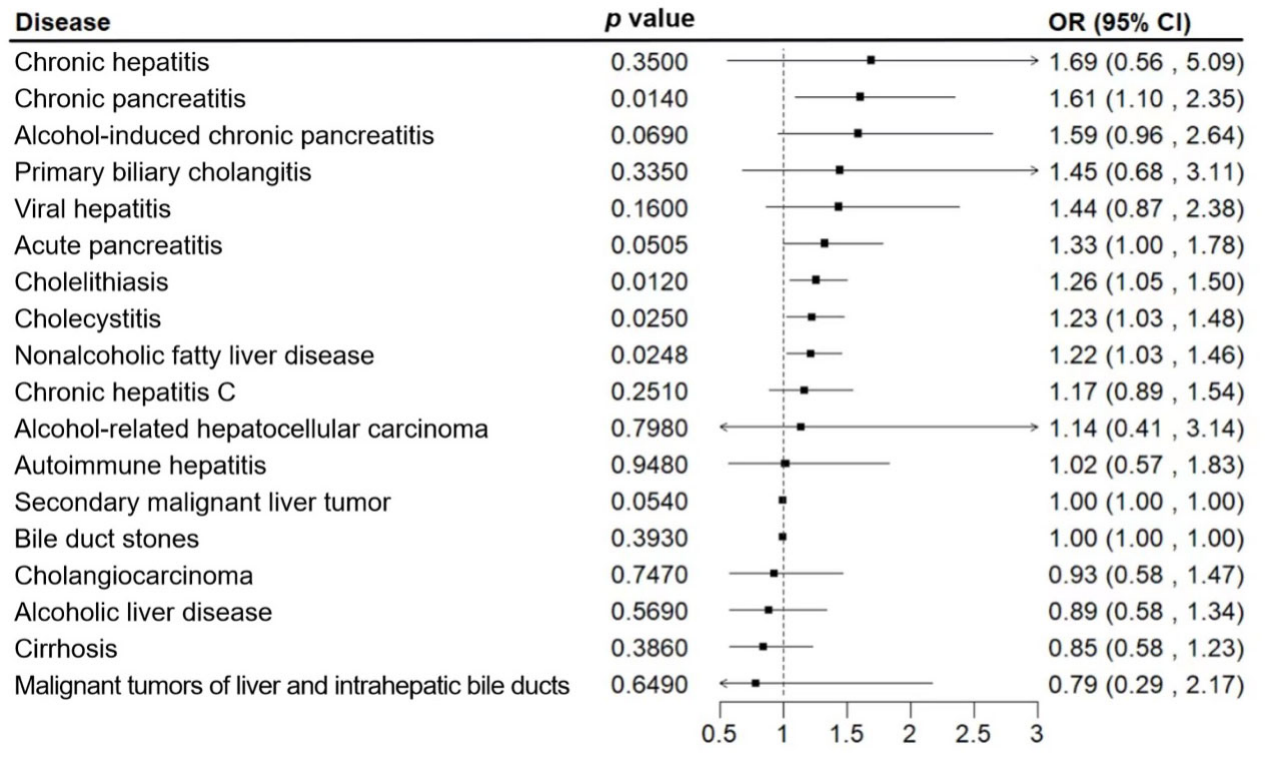
Associations of genetic liability to major depression with 18 hepatobiliary diseases. The estimates of hepatobiliary diseases were meta-analysis by combining estimates from the UK Biobank and the FinnGen study.

To verify the bidirectional causal relationship between depression and hepatobiliary diseases, we conducted a bidirectional MR analysis (**Figure 3**) and found that among the 18 hepatobiliary diseases studied, depression was positively correlated with 4 diseases, including non-alcoholic fatty liver disease (OR, 1.22; 95% CI, 1.03-1.46; *p*=0.0248), cholelithiasis (OR, 1.26; 95% CI, 1.05-1.50; *p*=0.0120), and chronic pancreatitis (OR, 1.61; 95% CI, 1.10-2.35; *p*=0.0140), cholecystitis (OR, 1.23; 95% CI, 1.03-1.48; *p*=0.0250), and after multiple corrections, these correlations still exist (**Figure 4**).

**Figure 5:**
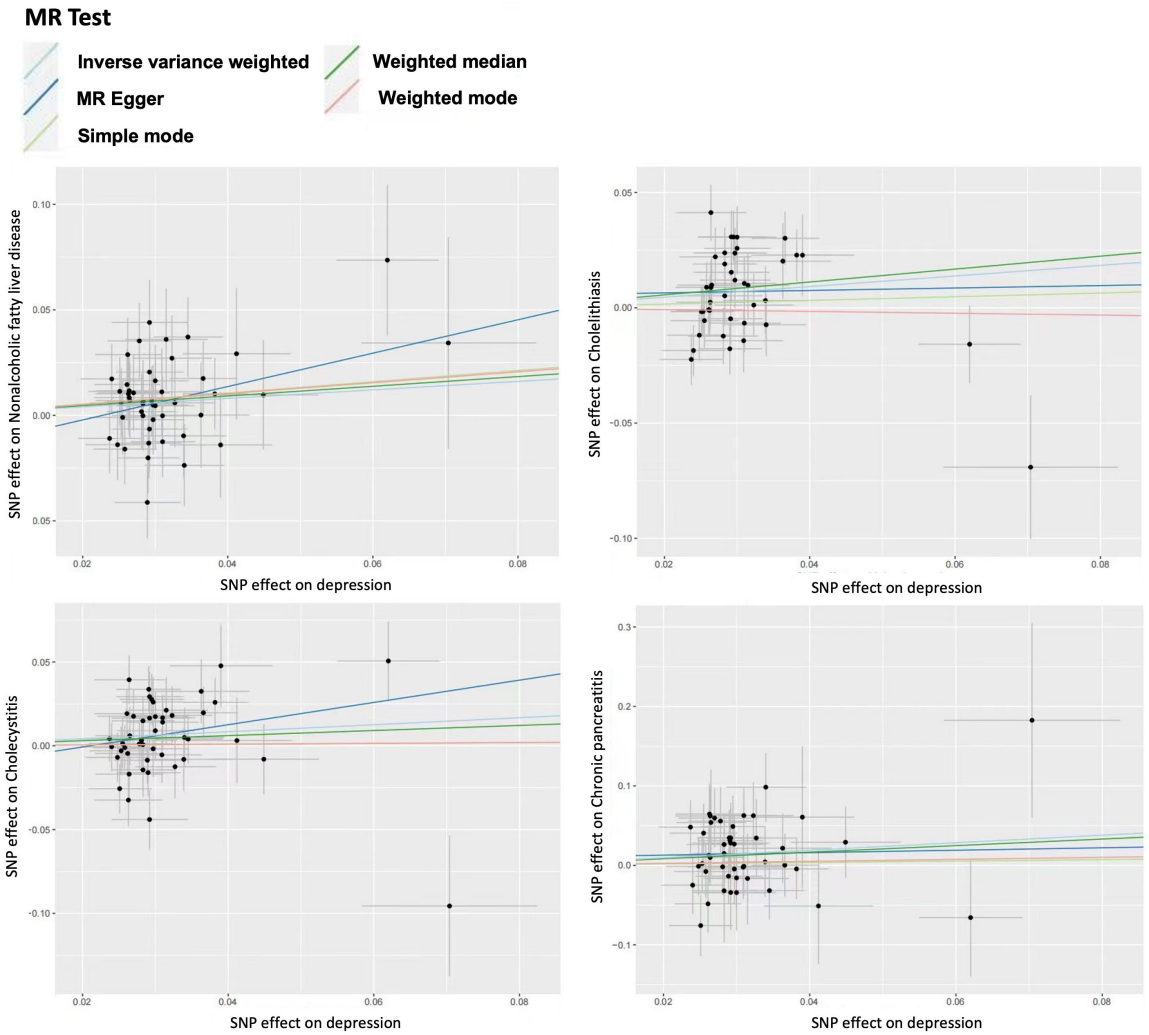
Scatter plot for univariable mendelian randomization analysis. Nonalcoholic fatty liver disease. Cholelithiasis. Cholecystitis. Chronic pancreatitis.

**Figure 6:**
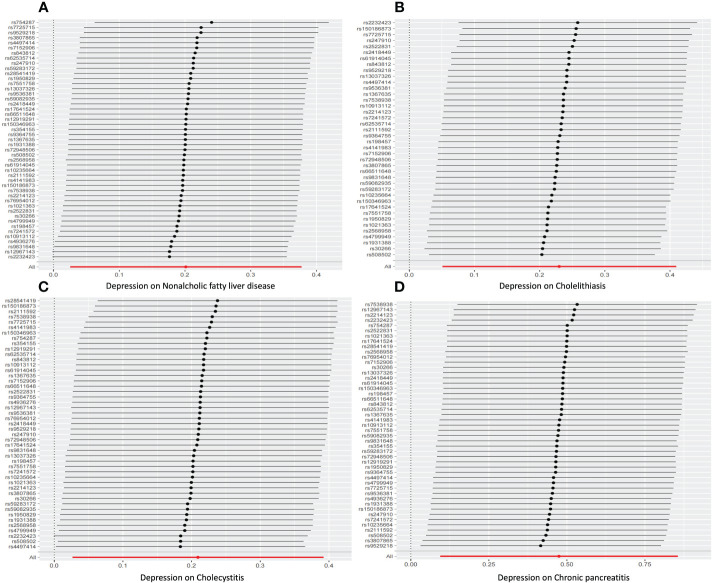
leave-one-out sensitivity analysis for Univariable mendelian randomization. **(A)** Nonalcoholic fatty liver disease. **(B)** Cholelithiasis. **(C)** Cholecystitis. **(D)** Chronic pancreatitis.

For sensitivity analysis, we used the Cochran’s Q test to evaluate the heterogeneity between individual genetic variation estimates, and the results showed heterogeneity (*p*<0.05) in the univariate analyses of depression-gallstones and depression- cholecystitis. Subsequently, we evaluated the level of multi efficacy using MR egger intercept, and the intercept value was used to estimate whether genetic variation significantly affects the results through pathways other than exposure (**Figure 5**). At the same time, we plotted a Leave-one-out plot (**Figure 6**). The results indicate that there is no significant level of pleiotropy in UVMR between depression and four hepatobiliary diseases, so we cautiously believe that the evaluation results using the IVW method are robust. To ensure the accuracy of the IVW results, two methods, Weighted media and MR Egger, were also used for validation (**Table 1**).

**Table 1:** Mendelian randomization results for the bidirectional associations between major depression and four hepatobiliary diseases.

Exposure	Outcome	Method	Beta	OR(95%CI)	*p* value	Cochran's Q	*p* value for pleiotropy*
Major depression	Nonalcoholic fatty liver disease	IVW	0.201	1.22 (1.03,1.46)	0.025a	0.642	
		MR-Egger	0.791	2.21(0.80,6.09)	0.134		0.254
		Weighted median	0.230	1.26(0.97,1.63)	0.079		
Major depression	Cholelithiasis gall stones	IVW	0.230	1.26 (1.05,1.50)	0.012a	0.000102	
		MR-Egger	0.053	1.05(0.41,2.71)	0.913		0.71
		Weighted median	0.280	1.32(1.08,1.63)	0.008		
Major depression	Cholecystitis	IVW	0.209	1.23 (1.03,1.48)	0.025a	0.017	
		MR-Egger	0.665	1.94(0.72,5.22)	0.194		0.363
		Weighted median	0.152	1.16(0.92,1.47)	0.198		
Major depression	Chronic pancreatitis	IVW	0.474	1.61 (1.10,2.35)	0.014a	0.734	
		MR-Egger	0.154	1.17(0.13,10.30)	0.89		0.771
		Weighted median	0.414	1.51(0.88,2.60)	0.134		
Nonalcoholic fatty liver disease	Major depression	IVW	-0.058	0.94 (0.86,1.04)	0.237	0.107	
		MR-Egger	-0.183	0.83(0.67,1.04)	0.244		0.346
		Weighted median	-0.064	0.94(0.87,1.02)	0.119		
Cholelithiasis gall stones	Major depression	IVW	-0.040	0.96 (0.90,1.02)	0.217	0.001	
		MR-Egger	-0.089	0.91(0.76,1.09)	0.338		0.57
		Weighted median	-0.045	0.96(0.89,1.02)	0.187		
Cholecystitis	Major depression	IVW	-0.022	0.98 (0.94,1.02)	0.291	0.487	
		MR-Egger	0.003	1.00(0.94,1.07)	0.92		0.333
		Weighted median	-0.003	1.00(0.95,1.05)	0.919		
Chronic pancreatitis	Major depression	IVW	0.039	1.04 (0.97,1.11)	0.255	0.241	
		MR-Egger	0.0006	1.00(0.81,1.24)	0.996		0.741
		Weighted median	0.024	1.02(0.95,1.10)	0.499		

CI, confidence interval; IVW, inverse-variance weighted; MR, Mendelian randomization; OR, odds ratio; *p value for pleiotropy based on MR-Egger intercept. ^a^Significant associations with p value < 0.05 after multiple testing.

In addition, to verify the bidirectional causal relationship between these four diseases and depression, we also conducted reverse MR analysis and the validation results showed that non-alcoholic fatty liver disease, cholelithiasis, chronic pancreatitis, and cholecystitis did not have a direct causal relationship with depression (*p*>0.05) (**Table 1**).

### Analysis of the role of mediators in depression and four types of hepatobiliary diseases

The genetic susceptibility to depression is significantly associated with a higher risk of waist-hip ratio, hypertension, and daytime nap. **Table 2** shows the results of multivariate MR analysis and the mediating effects of a single medium and a combination of three media. We noticed that napping (13.26%) and a combination of three mediators (35.8%) mediated the impact of depression on non-alcoholic fatty liver disease, and napping also had a certain mediating effect on the effects of depression on gallstones (22.37%) and cholecystitis (16.92%).

**Table 2:** Estimates of depression on hepatobiliary diseases mediated by potential mediators.

Hepatobiliary Dieases	Adjustment for Hypertension			Adjustment for Daytime nap			Adjustment for Waist-hip ratio			Adjustment for Hypertension, Daytime nap and Waist-hip ratio		
	OR (95% CI)	*p* value	Mediation effect (%)	OR (95% CI)	*p* value	Mediation effect(%)	OR (95% CI)	*p* value	Mediation effect(%)	OR (95% CI)	*p* value	Mediation effect(%)
**Nonalcoholic fatty** **liver disease**	1.22 (1.02,1.47)	0.026	8.48%	1.18 (0.99,1.41)	0.060	13.26%	1.12 (0.89,1.40)	0.334	8.38%	1.14 (0.92,1.41)	0.232	35.80%
**Cholelithiasis**	1.26 (1.04,1.51)	0.015	0.65%	1.27 (1.06,1.52)	0.009	22.37%	1.33 (1.10,1.60)	0.003	2.58%	1.33 (1.11,1.60)	0.002	-25.36%
**Cholecystitis**	1.29 (1.08,1.54)	0.004	-1.89%	1.27 (1.07,1.51)	0.006	16.92%	1.32 (1.09,1.59)	0.004	2.97%	1.30 (1.08,1.56)	0.006	-24.38%
**Chronic pancreatitis**	1.54 (1.05,2.26)	0.027	2.52%	1.69 (1.15,2.50)	0.008	6.50%	1.94 (1.25,3.01)	0.003	4.15%	1.98 (1.29,3.06)	0.002	-44.34%

OR, odds radio; CI, confidence interval.

## Discussion

We conducted a comprehensive MR analysis on the relationship between depression and 18 hepatobiliary diseases. We found that depression increases the risk of four hepatobiliary diseases (NAFLD, cholelithiasis, chronic pancreatitis, and cholecystitis). Multivariate MR analysis indicates that the association between depression and non-alcoholic fatty liver disease is mainly mediated by nap. Reverse MR analysis did not find any evidence to suggest that the four hepatobiliary diseases mentioned above are related to depression.

For the results of univariate analysis, many epidemiological studies have explored the relationship between depression and hepatobiliary diseases. The incidence of depression in patients with chronic liver disease is higher than that in the general population, including chronic hepatitis B, chronic hepatitis B, alcoholic liver disease, and non-alcoholic fatty liver disease (34). In a study exploring the clinical characteristics of NAFLD patients with severe depression, Tomeno et al. found that NAFLD patients with severe depression had significant changes in liver fat, higher NAFLD activity scores, transaminase, GGT, and ferritin levels (35). Depression is a psychological response to stress. Chronic psychological stress can activate the hypothalamic pituitary adrenal axis, promote the synthesis and secretion of glucocorticoids, lead to the accumulation of fat in liver cells and an increase in blood sugar concentration. The increase in blood sugar can induce insulin resistance, leading to liver lipid metabolism disorder and low-grade systemic inflammation, increasing inflammatory mediators such as IL-6 and TNF-a in the plasma. These inflammatory mediators can stimulate Kupffer cells and hepatic stellate cells, leading to liver inflammation and fibrosis changes, promoting the development of NAFLD towards NASH and liver fibrosis (36). Moreover, disturbances in liver lipid metabolism can also lead to abnormal cholesterol metabolism, resulting in an imbalance in the ratio of cholesterol to bile acids and phospholipids in bile, collectively leading to the occurrence of gallstones. Different studies have shown that patients with liver and gallbladder cancer typically experience considerable psychological distress, and about a quarter of patients suffer from depressive symptoms (37–39). Pancreatic diseases, especially pancreatitis, are considered potential risk factors for depression and anxiety (40, 41). Our univariate bidirectional MR analysis results indicate that depression increases the risk of NAFLD, chronic pancreatitis, cholecystitis, and gallstones, which confirms previous epidemiological studies from a causal perspective.

Our MR repeated the positive correlation between depression and non-alcoholic fatty liver disease and found that depression does indeed lead to an increase in the incidence of non-alcoholic fatty liver disease. However, there is no clear evidence to suggest that non-alcoholic fatty liver disease increases the risk of depression, which may be due to unmeasured confounding factors and differences caused by relatively small sample sizes. Most of the research on depression and liver diseases focuses on non-alcoholic fatty liver disease, and other types of liver diseases are rarely involved. Our MR study improved the causal relationship between depression and other liver diseases, and the results showed that depression does not have a causal relationship with chronic hepatitis, viral hepatitis, chronic hepatitis C, alcohol related hepatocellular carcinoma, autoimmune hepatitis, secondary malignant liver tumors, hepatobiliary carcinoma, alcoholic liver disease, cirrhosis, liver and intrahepatic bile duct malignant tumors (*p*>0.05). Although the relationship between depression and these liver diseases is negative, this is also a new discovery, and further validation of the relationship requires more data samples and clinical trials. In addition, Cochran’s Q test for heterogeneity found heterogeneity (p<0.05) in the UVMR analysis of depression gallstones and depression cholecystitis. Since we used random effects IVW as the main result, heterogeneity was acceptable (42). However, heterogeneity can affect the accuracy of subsequent mediation effect calculations, which is one of the limitations of this study. Further prospective cohort studies are needed to replicate our analysis.

Mediation Mendelian randomization is used to evaluate the mediating effect of a mediator variable between exposure factors and outcome variables. In this study, we quantified the mediating role of waist to hip ratio, nap time, and hypertension in the risk of depression and hepatobiliary diseases. NAFLD has traditionally been considered a consequence of MetS. However, NAFLD and MetS components, especially type 2 diabetes, hypertension and cardiovascular disease are closely related. In addition, a logistic regression analysis has determined that the waist to hip ratio can serve as a relatively simple indicator for diagnosing NAFLD. In our study, we found that hypertension and waist to hip ratio have a weak mediating effect on depression in four hepatobiliary diseases. However, napping has a relatively strong mediating effect on non-alcoholic fatty liver disease, gallstones, and cholecystitis in depression. In general, adequate sleep plays a vital role in restoring physical strength and regulating body and mind, and is one of the conditions for maintaining health (43). Most people do not realize that siesta may be closely related to chronic diseases. Some studies have found that subjects with chronic metabolic or cardiovascular diseases take siesta more frequently than their healthy peers. Regardless of the length of sleep, siesta is related to diabetes, obesity, epidemic stroke and myocardial infarction in elderly women (27). Regarding the relationship between napping and depression, studies have shown that nappers are slightly more likely to suffer from depression than non nappers, and daytime napping may be a secondary result or symptom of poor health and sleep disorders, which may be an independent risk factor for depression (44). In addition, an MR analysis study on identifying modifiable factors for preventing depression found that the trend of daytime napping in adults seems to increase the risk of depression (45), especially when napping for more than an hour (46). According to multivariate MR analysis results, nap time to some extent mediates the impact of depression on non-alcoholic fatty liver, cholelithiasis, and cholecystitis. If depression does not increase nap time, it may not have an impact on these diseases. Therefore, our MR analysis is of great significance for clinical guidance of doctors in treating patients with depression.

Our research results mainly rely on MR analysis, which can minimize the influence of confounding factors and ensure the accuracy of the results. At the same time, multivariate MR can be used to verify the mediating role of multiple mediators in causal relationships. However, this study also has certain limitations. Due to the lack of complete instrumental variables for hepatobiliary diseases, the definition of depression is also different, mainly targeting the European population. Therefore, the study cannot cover other groups. In our study, we used large-scale genetic data to obtain instrumental variables, both the exposure and outcome datasets used research data from the UK Biobank, which may bias MR estimates towards observational associations. In the reverse MR analysis, due to the set screening criteria with a genome-wide significance threshold P<5×10^-8^, we obtained a relatively small number of SNPs, and further research is needed to verify this. In addition, due to the updated naming and definition of NAFLD, this study has certain limitations. “Metabolic Dysfunction Associated Fatty Liver Disease (MAFLD)” is a term proposed in 2020, referring to fatty liver disease associated with systemic metabolic disorders (47). In 2023, three major multinational liver associations proposed that the term “Metabolic Dysfunction Associated Fatty Liver Disease (MASLD)” should replace the term “NAFLD” (48). With the update of standards and the differences between the two, our research should collect new data and conduct further research based on the new standards. To ensure the accuracy of the results, we conducted multiple sensitivity analyses and found that there was no pleiotropy in the research results. Furthermore, the association between exposure factors and outcome variables derived from UK Biobank was replicated in the FinnGen study, which reduced the bias in the results caused by sample overlap. However, despite conducting multiple sensitivity analyses, we cannot completely rule out heterogeneity and horizontal pleiotropy, as our instrument may still be affected by unmeasurable confounding or other sources of bias.

In summary, our MR study has shown that depression is associated with an increased risk of four hepatobiliary diseases, and no evidence has been found to suggest that these four hepatobiliary diseases are associated with depression. Among the effects of depression on four types of hepatobiliary diseases, napping to a greater extent mediates the relationship between waist to hip ratio and hypertension.

## Conclusion

In summary, our research has shown that depression is a susceptibility factor for non-alcoholic fatty liver disease, and the causal effect of genetic susceptibility to depression on non-alcoholic fatty liver disease is mediated by waist to hip ratio, hypertension, and napping. This finding emphasizes the role of depression in non-alcoholic fatty liver disease and suggests clinical attention to the risk of depression in non-alcoholic fatty liver disease patients.

## Data availability statement

The datasets presented in this study can be found in online repositories. The names of the repository/repositories and accession number(s) can be found in the article/**Supplementary Material.**

## Ethics statement

The studies involving humans were approved by The Mendelian randomization analysis of this study was based on publicly available data and obtained ethical approval. The studies were conducted in accordance with the local legislation and institutional requirements. The participants provided their written informed consent to participate in this study.

## Author contributions

YK: Writing – original draft, Software, Formal Analysis, Data curation. ZY: Writing – review & editing, Supervision, Data curation, Conceptualization. LR: Writing – original draft, Validation, Formal Analysis. LZ: Writing – review & editing, Methodology, Data curation. JZ: Writing – review & editing, Formal Analysis, Data curation. YQ: Writing – review & editing, Visualization, Supervision. DL: Writing – review & editing, Visualization, Supervision, Funding acquisition, Conceptualization.
