# Supplementary material for: Depression and hepatobiliary diseases: a bidirectional Mendelian randomization study
Source: Front Psychiatry. 2024 Mar 26;15:1366509. doi: 10.3389/fpsyt.2024.1366509 (PMC11002219; doi:10.3389/fpsyt.2024.1366509)
Supplement: Supplementary file 1 [file Table_1.docx]

**Supplementary materials**

**Table S1. Information of included studies and consortia.**

| **Exposure/Mediator/Outcome** | **GWAS ID** |
| --- | --- |
| major depression | ieu-b-102 |
| nonalcoholic fatty liver disease | ebi-a-GCST90091033 |
| viral hepatitis | finn-b-AB1_VIRAL_HEPATITIS |
| malignant tumors of liver and bile ducts | finn-b-C3_LIVER_INTRAHEPATIC_BILE_DUCTS |
| cirrhosis | finn-b-CIRRHOSIS_BROAD |
| cholelithiasis | ebi-a-GCST90013889 |
| cholecystitis | ebi-a-GCST90018818 |
| primary biliary cholangitis | ebi-a-GCST003129 |
| autoimmune hepatitis | ebi-a-GCST90018785 |
| cholangiocarcinoma | ebi-a-GCST90018803 |
| bile duct stones | ukb-b-8268 |
| chronic hepatitis C | ebi-a-GCST90018805 |
| chronic hepatitis | finn-b-CHRONHEP_NAS |
| Secondary malignant liver tumor | ukb-b-16713 |
| acute pancreatitis | finn-b-K11_ACUTPANC |
| alcoholic liver disease | finn-b-K11_ALCOLIV |
| alcohol-induced chronic pancreatitis | finn-b-ALCOPANCCHRON |
| chronic pancreatitis | ebi-a-GCST90018821 |
| alcohol-related hepatocrllular carcinoma | ebi-a-GCST90092003 |
| Hypertension | finn-b-I9_HYPTENS |
| Daytime nap | ebi-a-GCST011494 |
| Waist-hip ratio | ebi-a-GCST90029009 |
