# Supplementary material for: Depression and hepatobiliary diseases: a bidirectional Mendelian randomization study
Source: Front Psychiatry. 2024 Mar 26;15:1366509. doi: 10.3389/fpsyt.2024.1366509 (PMC11002219; doi:10.3389/fpsyt.2024.1366509)
Supplement: Supplementary file 2 [file Table_2.docx]

**Supplementary materials**

**Table S2. Genetic Variants (n=50) of Depression Used in MR Analyses.**

| **SNP** | **effect allele** | **other allele** | **Eaf** | **Beta** | **Se** | **Pval** | **F** |
| --- | --- | --- | --- | --- | --- | --- | --- |
| rs7551758 | G | T | 0.5329 | 0.0283 | 0.0043 | 5.11E-11 | 43.31 |
| rs2568958 | A | G | 0.6042 | 0.0382 | 0.0044 | 2.90E-18 | 75.37 |
| rs10913112 | T | C | 0.378 | -0.0262 | 0.0045 | 4.53E-09 | 33.90 |
| rs17641524 | T | C | 0.2101 | -0.03 | 0.0053 | 1.50E-08 | 32.04 |
| rs354155 | C | G | 0.0923 | -0.0449 | 0.0075 | 1.75E-09 | 35.84 |
| rs7538938 | C | T | 0.5599 | 0.0251 | 0.0043 | 7.29E-09 | 34.07 |
| rs4141983 | C | T | 0.326 | -0.0264 | 0.0046 | 9.69E-09 | 32.94 |
| rs2111592 | A | G | 0.3141 | 0.0263 | 0.0046 | 1.35E-08 | 32.69 |
| rs72948506 | A | G | 0.2975 | 0.0265 | 0.0047 | 1.71E-08 | 31.79 |
| rs35469634 | G | A | 0.5774 | -0.0241 | 0.0044 | 3.28E-08 | 30.00 |
| rs843812 | A | G | 0.4117 | 0.0248 | 0.0044 | 1.41E-08 | 31.77 |
| rs9831648 | T | G | 0.7739 | -0.0292 | 0.0052 | 1.59E-08 | 31.53 |
| rs66511648 | C | T | 0.284 | 0.0297 | 0.0048 | 6.03E-10 | 38.29 |
| rs76954012 | A | T | 0.0931 | 0.0412 | 0.0074 | 2.41E-08 | 31.00 |
| rs30266 | A | G | 0.3271 | 0.0366 | 0.0046 | 1.43E-15 | 63.31 |
| rs247910 | G | A | 0.457 | 0.0237 | 0.0043 | 4.71E-08 | 30.38 |
| rs7725715 | A | G | 0.5343 | 0.029 | 0.0043 | 1.61E-11 | 45.48 |
| rs150186873 | C | A | 0.0327 | 0.0704 | 0.012 | 4.51E-09 | 34.42 |
| rs2232423 | G | A | 0.1056 | -0.062 | 0.007 | 1.14E-18 | 78.45 |
| rs9364755 | G | A | 0.2262 | 0.0283 | 0.0051 | 3.49E-08 | 30.79 |
| rs2214123 | G | A | 0.6466 | -0.0261 | 0.0045 | 8.56E-09 | 33.64 |
| rs2876520 | G | C | 0.4688 | 0.026 | 0.0043 | 2.24E-09 | 36.56 |
| rs2522831 | C | T | 0.4739 | 0.024 | 0.0043 | 2.11E-08 | 31.15 |
| rs4730387 | A | T | 0.4659 | 0.0238 | 0.0043 | 4.12E-08 | 30.63 |
| rs150346963 | T | C | 0.4118 | 0.0283 | 0.0044 | 1.16E-10 | 41.37 |
| rs3807865 | A | G | 0.4105 | 0.031 | 0.0044 | 1.09E-12 | 49.64 |
| rs10235664 | C | T | 0.2529 | -0.027 | 0.0049 | 4.68E-08 | 30.36 |
| rs59082935 | T | C | 0.1342 | 0.0363 | 0.0066 | 3.07E-08 | 30.25 |
| rs62535714 | A | G | 0.1639 | 0.0339 | 0.0058 | 4.69E-09 | 34.16 |
| rs1931388 | G | A | 0.4042 | -0.0295 | 0.0044 | 1.68E-11 | 44.95 |
| rs59283172 | A | G | 0.1081 | -0.039 | 0.007 | 2.41E-08 | 31.04 |
| rs2418449 | C | T | 0.281 | -0.0281 | 0.0048 | 4.25E-09 | 34.27 |
| rs1021363 | G | A | 0.6434 | -0.03 | 0.0045 | 2.29E-11 | 44.44 |
| rs198457 | T | C | 0.1886 | -0.0315 | 0.0056 | 1.90E-08 | 31.64 |
| rs4497414 | C | T | 0.44 | 0.0291 | 0.0044 | 2.93E-11 | 43.74 |
| rs4936276 | C | G | 0.622 | 0.0278 | 0.0044 | 3.57E-10 | 39.92 |
| rs61914045 | A | G | 0.2034 | 0.0309 | 0.0054 | 7.96E-09 | 32.74 |
| rs9529218 | T | C | 0.2031 | -0.034 | 0.0054 | 2.23E-10 | 39.64 |
| rs9536381 | T | C | 0.3259 | 0.0255 | 0.0046 | 2.62E-08 | 30.73 |
| rs508502 | T | C | 0.2992 | -0.0264 | 0.0048 | 3.56E-08 | 30.25 |
| rs1950829 | G | A | 0.5173 | -0.0297 | 0.0043 | 4.74E-12 | 47.71 |
| rs754287 | A | T | 0.3664 | -0.0289 | 0.0045 | 1.31E-10 | 41.24 |
| rs7152906 | C | T | 0.5196 | 0.0258 | 0.0043 | 1.87E-09 | 36.00 |
| rs28541419 | G | C | 0.2308 | -0.0292 | 0.0052 | 1.76E-08 | 31.53 |
| rs12919291 | C | G | 0.1884 | 0.0327 | 0.0055 | 3.09E-09 | 35.35 |
| rs4799949 | T | C | 0.6684 | -0.0292 | 0.0046 | 1.40E-10 | 40.29 |
| rs12967143 | C | G | 0.7012 | -0.0345 | 0.0047 | 2.53E-13 | 53.88 |
| rs7241572 | A | G | 0.2047 | 0.0323 | 0.0054 | 2.43E-09 | 35.78 |
| rs1367635 | C | T | 0.5148 | 0.0253 | 0.0043 | 4.35E-09 | 34.62 |
| rs13037326 | T | C | 0.2597 | 0.031 | 0.0049 | 2.40E-10 | 40.02 |
